# A Thermally Controlled Ultra-Wideband Wide Incident Angle Metamaterial Absorber with Switchable Transmission at the THz Band

**DOI:** 10.3390/nano15050404

**Published:** 2025-03-06

**Authors:** Liansheng Wang, Fengkai Xin, Quanhong Fu, Dongyan Xia

**Affiliations:** 1 New Energy and Intelligent Vehicle Department, Sanya University, Sanya 572022, China; fengkaixin@sanyau.edu.cn; 2 School of Physical Science and Technology, Northwestern Polytechnical University, Xi’an 710129, China; fuquanhong@nwpu.edu.cn; 3 Finance and Economics Department, Sanya University, Sanya 572022, China; dongyanxia@sanyau.edu.cn

**Keywords:** metamaterial absorber, VO_2_ thin film, thermally controlled, wide incident angle, transmission, ultra-wideband

## Abstract

We demonstrate a thermally controlled ultra-wideband wide incident angle metamaterial absorber with switchable transmission at the THz band in this paper. The underlying hybrid structure of FSS-VO_2_ thin films make them switchable between absorption mode and transmission mode by controlling the temperature. It can achieve ultra-wideband absorption with above 90% absorption from 1 THz to 10 THz and exhibits excellent absorption performance under a wide range of incident and polarization angles at a high temperature (80 °C). At room temperature (27 °C), it acts in transmission mode with a transmission coefficient of up to 60% at 3.1278 THz. The transmission region is inside the absorption band, which is very important for practical applications. The metamaterial absorber has the advantage of easy fabrication, an ultra-wideband, a wide incident angle, switchable multi-functions, and passivity with wide application prospects on terahertz communication and radar devices.

## 1. Introduction

Metamaterials are a type of artificially structured material that exhibits numerous exotic effects unattainable in natural materials [1,2,3] and operates across a broad electromagnetic spectral range [4,5]. These exotic effects have driven the emergence of numerous metamaterial devices, such as gradient index lenses [6,7], modulators for incident waves [8,9,10,11,12,13,14,15], and compact waveguides [16]. Metamaterial absorbers are a significant branch of metamaterials, which have the advantages of wideband absorption, easy fabrication, light weight, and so on [17,18]. Due to their outstanding advantages, metamaterial absorbers hold significant application value in fields such as wireless communication, electromagnetic shielding and cloaking, electromagnetic sensing, and so on. Terahertz communication possesses unparalleled advantages, such as ultra-wideband capabilities, that modern communication technologies cannot match. The advancement of terahertz technology makes it highly probable that terahertz communication will become a reality in the near future.

In recent years, tunable metamaterials, which are based on phase-change materials, have attracted extensive attention from researchers due to their wide potential for practical application [19,20,21,22]. In future terahertz communication devices, metamaterial absorbers with switchable transmission windows will be strongly demanded, as they can transmit signals in predetermined frequency bands and absorb signals in out-of-band frequencies. Vanadium dioxide (VO_2_) is a thermally controlled insulator–metal phase transition material. Regarding the research aspect of terahertz metamaterial absorbers with switchable transmission windows, a metal frequency selective surface with loaded VO_2_ thin films has almost been used to constitute the underlying layer of a metamaterial absorber [23,24,25,26]. At room temperature (27 °C), VO_2_ thin film exists in an insulating state. In this state, the underlying layer of the metamaterial absorber adopts a frequency selective surface (FSS) structure, allowing transmission of electromagnetic waves within specific frequencies. When heated to a critical temperature (80 °C), VO_2_ thin film undergoes a phase transition to a metallic state. In this metallic phase, the underlying layer of the metamaterial absorber transforms into a fully metallic structure, enabling effective absorption of incident electromagnetic waves. The abovementioned terahertz metamaterial absorbers with switchable transmission windows exhibit certain limitations in their fabrication process, absorption bandwidth, in-band transmission and out-of-band absorption performances, and so on [23,24,25,26].

In this paper, we present a thermally controlled ultra-wideband wide incident angle metamaterial absorber with switchable transmission at the THz band based on a VO_2_ thin film. It can achieve ultra-wideband absorption with above 90% absorption from 1 THz to 10 THz and exhibits excellent absorption performance under a wide range of incident and polarization angles at a high temperature (80 °C). At room temperature (27 °C), it acts in transmission mode with a transmission coefficient of up to 60% at 3.1278 THz. It has the advantages of easy fabrication, an ultra-wideband, switchable multi-functions, and passivity with wide application prospects on terahertz communication and radar devices.

## 2. Model Design

**Figure 1:**
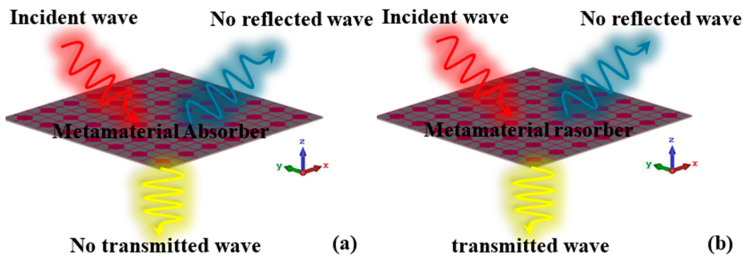
A schematic of our designed metamaterial absorber: (**a**) at a high temperature (80 °C); (**b**) at room temperature (27 °C).

**Figure 2:**
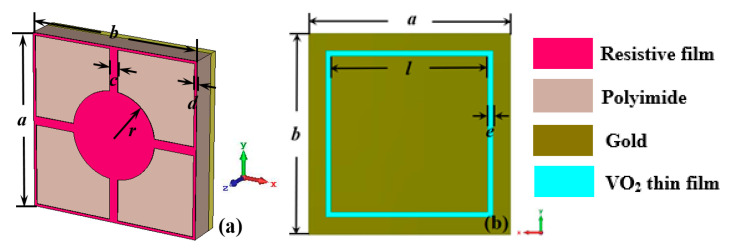
Geometry of a unit cell: (**a**) perspective view; (**b**) back view.

A schematic of our designed thermally controlled ultra-wideband wide incident angle metamaterial absorber with switchable transmission at the THz band is depicted in Figure 1. A typical unit cell, illustrated in Figure 2, consists of a resistive film resonator with a sheet resistance of 1800 Ω/□ at the top layer, a gold frequency selective surface with a loaded VO_2_ thin film at the bottom, and a polyimide (permittivity *ε*_r_ = 3.5, tangent value of loss angle tanδ = 0.0027) as the substrate. The parameter-optimized dimensions of the unit cell in Figure 2 are *a* = *b* = 20 μm, *c* = 1 μm, *d* = 0.3 μm, *r* = 5 μm, *l* = 16 μm, and *e* = 0.5 μm.

VO_2_ is an excellent phase-change material that exhibits an insulating state at room temperature and transitions to the metallic state when the ambient temperature exceeds 68 °C. At the THz band, according to the Bruggeman effective medium theory, the permittivity of VO_2_ can be expressed as [23,27,28]

(1) $$\begin{array}{l} \varepsilon_{c}=\frac{1}{4}\{\varepsilon_{i}(2-3\phi )+\varepsilon_{m}(3\phi -1)\}+\\ \frac{1}{4}\{\sqrt{{\left[\varepsilon_{i}(2-3\phi )+\varepsilon_{m}(3\phi -1)\right]}^{2}+8\varepsilon_{i}\varepsilon_{m}}\} \end{array}$$

where $\varepsilon_{i}$ and $\varepsilon_{m}$ are the permittivity of the insulating and metallic states, respectively, and ϕ is the volume fraction of the metallic component, which is temperature-dependent and varies with external temperature changes. The permittivity of the insulating component is $\varepsilon_{i}=9$, while the permittivity of the metallic component can be described by the Drude model [23,27,28]:

(2) $$\varepsilon_{r}(\omega )=\varepsilon_{\infty }-\frac{\omega_{p}^{2}(\sigma )}{\omega_{2}+i\gamma \omega }$$

(3) $$\omega_{p}^{2}(\sigma_{{\mathrm{VO}}_{2}})=\frac{\sigma_{{\mathrm{VO}}_{2}}}{\sigma_{0}}\omega_{p}^{2}(\sigma_{0})$$

where $\varepsilon_{\infty }=9$ is the high-frequency permittivity; *γ* = 5.75 × 10^13^ rad/s is the collision frequency; and $\omega_{p}(\sigma_{{\mathrm{VO}}_{2}})$ is the plasma frequency, which depends on conductivity $\sigma_{0}$. The plasma frequency is given by $\omega_{p}(\sigma_{0})=1.4\times 10^{15}\;\mathrm{rad}/s$ with $\sigma_{0}=3\times 10^{15}\;S/m$. During the temperature increase, the internal components of VO_2_ do not undergo phase transitions simultaneously, meaning that VO_2_ coexists in both the metallic and dielectric states. The volume fraction of the metallic component ϕ(T), as a function of temperature *T*, can be expressed as [23]

(4) $$\phi (T)=1-\frac{1}{1+\exp[(T-T_{0})/\Delta T]}$$

where *T*_0_ = 68 °C is the critical temperature at which the phase transition occurs and Δ*T* = 2 °C represents the transition temperature range. Consequently, the change in the volume fraction of the metallic component affects the conductivity of VO_2_, which can be expressed as [29]

(5) $$\sigma_{{\mathrm{VO}}_{2}}=-i\varepsilon_{0}\omega (\varepsilon_{c}-1)$$

**Figure 3:**
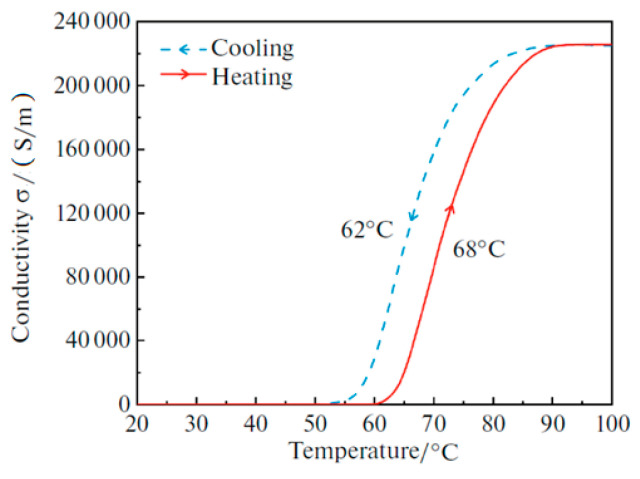
The conductivity of VO_2_ during heating and cooling processes.

As shown in Figure 3 [23], the VO_2_ conductivity changed from zero to 2.2 × 105 S/m as the temperature underwent a change. To describe the phase transition process of VO_2_, the conductivity at room temperature (27 °C) and a high temperature (80 °C) was calculated using Equations (1)–(5), yielding values of 20 S/m and 2 × 10^5^ S/m, respectively.

During the simulation and calculation process of our design, the VO_2_ thin film is set as an insulating medium with permittivity *ε*_i_ = 9 and conductivity *σ* = 20 S/m at room temperature (27 °C), while at a high temperature (80 °C), the VO_2_ thin film is set as a metal with conductivity *σ* = 2 × 10^5^ S/m.

The commercial software Microwave studio CST 2016 was used for the full-wave simulation of the designed metamaterial absorber. Throughout the simulation process, the boundary conditions for the *x* and *y* directions were set as a unit cell and the *z*-direction was set as open. The All + Floquet ports were used to simulate the incoming and outgoing waves. The electromagnetic parameters were calculated using a frequency-domain electromagnetic solver.

## 3. Results

**Figure 4:**
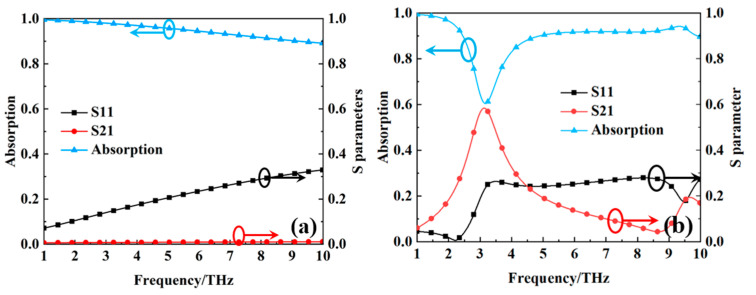
The absorption, transmission coefficient, and reflection coefficient of the metamaterial absorber: (**a**) the VO_2_ thin film is in the metallic phase at a high temperature of 80 °C; (**b**) the VO_2_ thin film is in the insulating phase at a room temperature of 27 °C.

Figure 4 shows the absorption, transmission coefficient, and reflection coefficient of the metamaterial absorber when the VO_2_ thin film is in the metallic phase at a high temperature of 80 °C and in the insulating phase at a room temperature of 27 °C. As can be seen from Figure 4, the absorption is over 90% in the 1–10 THz frequency range, with a relative bandwidth of 163% when the VO_2_ thin film is in the metallic phase at a high temperature of 80 °C, demonstrating ultra-broadband absorption characteristics. When the VO_2_ thin film is in the insulating phase at a room temperature of 27 °C, a transmission peak at 3.1278 THz appears, with the transmission coefficient reaching 60%, while maintaining over 90% absorption in the out-of-band regions of 1–2.4 THz and 4.6–10 THz. This indicates that the metamaterial absorber exhibits in-band transmission and out-of-band absorption properties, which play a significant role in practical applications. A comparison of the performance of our metamaterial absorber with the works in Refs. [23,24,25,26] is shown in Table 1. It can be seen from Table 1 that our designed metamaterial absorber has the advantages of easy fabrication and ultra-wideband absorption. While the transmission coefficient shows no inherent advantage, we can use the method of enhancing the incident signal’s power to effectively yield the required transmitted signal.

**Figure 5:**
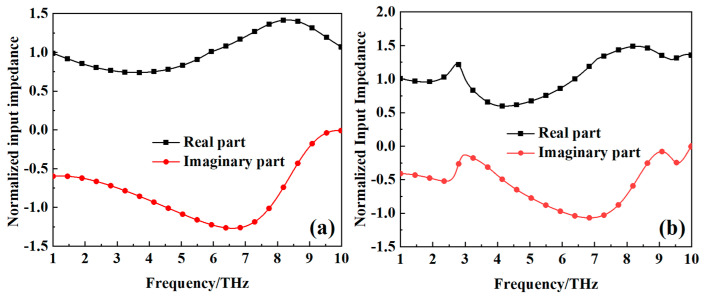
The normalized input impedance of the metamaterial absorber: (**a**) the VO_2_ thin film is in the metallic phase at a high temperature of 80 °C; (**b**) the VO_2_ thin film is in the insulating phase at a room temperature of 27 °C.

When the bottom layer of the metamaterial absorber is a metallic ground plane, the transmittance of the incident electromagnetic wave is effectively zero. Under this circumstance, the absorption can be quantified by the formula A(ω)=1−R(ω), where $R(\omega )={\left|S_{11}(\omega )\right|}^{2}$ denotes reflectivity. Perfect absorption is attainable when the reflectivity R(ω) approaches zero. The prerequisite for achieving zero reflectivity is that the normalized input impedance *Z*r (defined as $Zr(\omega )=Z(\omega )/Z_{0}$, where Z(ω) is the input impedance of the metamaterial absorber and $Z_{0}$ is the impedance of free space) must be unity. Figure 5 illustrates the normalized input impedance, derived via the scattering parameter method [30], for the metamaterial absorber when the VO_2_ thin film is in the metallic phase at a high temperature of 80 °C and in the insulating phase at a room temperature of 27 °C. As depicted in Figure 5a, the real component of the normalized input impedance approximates unity within the frequency range of 1 to 10 THz when the VO_2_ thin film is in the metallic phase at a high temperature of 80 °C. This condition implies minimal reflection of incident waves by the metamaterial absorber, thereby enhancing absorption. The presence of the negative imaginary component in the normalized input impedance suggests the significant loss of incident waves within the metamaterial absorber. As can be seen from Figure 5b, when the VO_2_ thin film is in the insulating phase at a room temperature of 27 °C, the real part of the normalized input impedance of the metamaterial absorber is also close to one within the range of 1–10 THz. This indicates that the incident electromagnetic wave enters the intermediate medium without loss. However, the square slot filled with VO_2_ thin films at the bottom metal plate allow for the transmission of incident electromagnetic waves at certain frequency. Additionally, Figure 5b shows that when the VO_2_ thin film is in the insulating phase at a room temperature of 27 °C, the imaginary part of the normalized input impedance of the metamaterial absorber is also negative within the range of 1–10 THz. This suggests that the incident electromagnetic wave experiences loss in the intermediate medium. However, the imaginary part of the normalized input impedance is relatively large at 3.1278 THz, indicating that the incident electromagnetic wave at 3.1278 THz possesses a certain transmission capability. This observation further corroborates the results shown in Figure 4b.

**Figure 6:**
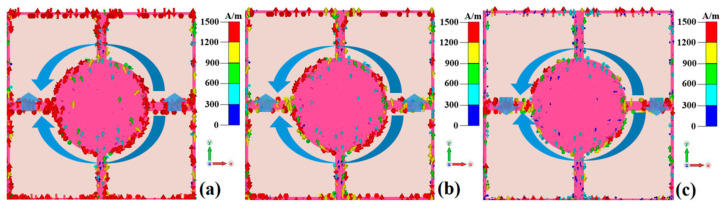
The top-layer surface current of a metamaterial absorber when the VO_2_ thin film is in the metallic phase at a high temperature (80 °C): (**a**) 3 THz; (**b**) 5 THz; (**c**) 7 THz.

**Figure 7:**
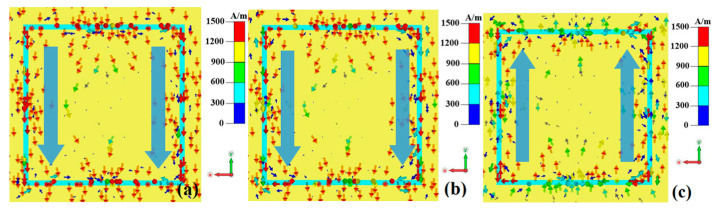
The bottom-layer surface current of a metamaterial absorber when the VO_2_ thin film is in the metallic phase at a high temperature (80 °C): (**a**) 3 THz; (**b**) 5 THz; (**c**) 7 THz.

**Figure 8:**
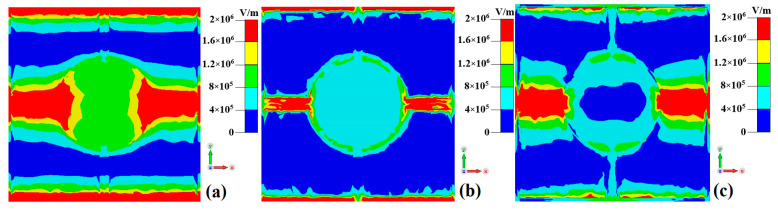
The electric field distributions of the metamaterial absorber when the VO_2_ thin film is in the metallic phase at a high temperature (80 °C): (**a**) 3 THz; (**b**) 5 THz; (**c**) 7 THz.

To further investigate the wideband absorption mechanism of the metamaterial absorber when the VO_2_ thin film is in the metallic phase at a high temperature (80 °C), the surface current and electric field distributions of the metamaterial absorber are monitored at 3 THz, 5 THz, and 7 THz, as shown in Figure 6, Figure 7 and Figure 8.

From Figure 6, it can be observed that the electric field component of the incident electromagnetic wave induces surface currents on the top-layer resonant unit of the metamaterial absorber. These currents are primarily concentrated along the upper and lower edges of the top-layer resistive film disk and the left–right connecting lines. Specifically, the surface currents on the upper and lower edges of the resistive film disk flow parallel to the left, leading to charge accumulation on the left and right sides of the disk, thereby forming electric resonance. Meanwhile, the surface currents on the left and right connecting lines flow upward at 3 THz and 5 THz but downward at 7 THz. This parallel current flow also causes charge accumulation at the upper and lower sections of the connecting lines, further contributing to electric resonance. These results indicate that the metamaterial absorber generates electric resonance under excitation of the electric field component of the incident electromagnetic wave [31].

As shown in Figure 7, the magnetic field component of the incident electromagnetic wave penetrates the intermediate dielectric layer and induces surface currents on the bottom metal plate. At 3 THz and 5 THz, the surface currents flow downward, while at 7 THz, they flow upward. This direction is opposite to that of the surface currents on the left–right connecting lines of the top-layer resistive film, forming closed current loops and thereby generating magnetic resonance. Figure 6 and Figure 7 collectively demonstrate that the metamaterial absorber simultaneously exhibits both electric and magnetic resonances under the incident electromagnetic wave, resulting in perfect absorption of the electromagnetic wave [31].

The presence of the top-layer resistive film enables the metamaterial absorber to act as a stable resonant circuit structure. Consequently, the impedance of the metamaterial absorber closely matches that of free space over a broad frequency range near the resonance, facilitating ultra-wideband absorption of incident waves [32].

As shown in Figure 8, the electric field in the metamaterial absorber under the incident electromagnetic wave is predominantly concentrated along the left and right connecting lines between the central disk and the outer square ring of the top-layer resistive film resonant unit. This observation aligns with the previous surface current distribution results of the top-layer resistive film resonant unit, as shown in Figure 6.

**Figure 9:**
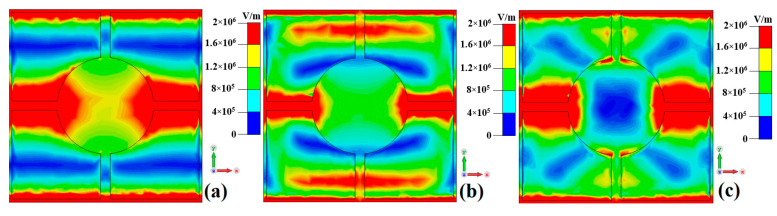
The electric field distributions of the metamaterial absorber when the VO_2_ thin film is in the insulating phase at a room temperature of 27 °C: (**a**) 1 THz; (**b**) 3.1278 THz; (**c**) 10 THz.

Figure 9 shows the electric field distributions of the metamaterial absorber at 1 THz, 3.1278 THz, and 10 THz when the VO_2_ thin film is in the insulating phase at a room temperature of 27 °C. As can be seen from Figure 9, the electric field distributions at 1 THz, 3.1278 THz, and 10 THz exhibit similarities to those when the VO_2_ thin film is in the metallic phase. However, the electric field intensity at a transmission frequency of 3.1278 THz is significantly lower compared to those at 1 THz and 10 THz. This phenomenon primarily occurs because the incident wave at 3.1278 THz possesses a certain transmission capability.

**Figure 10:**
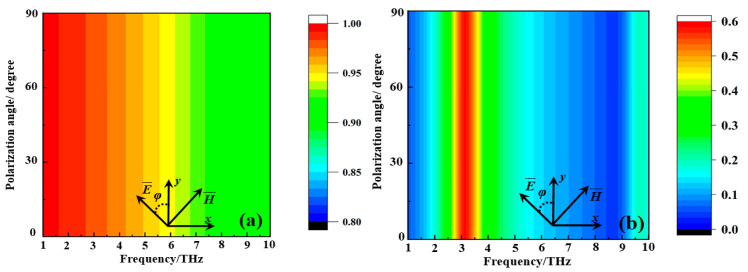
The absorption and transmission coefficient of the metamaterial absorber under different polarization angles when the VO_2_ film is in high-temperature (80 °C) and room-temperature (27 °C) environments: (**a**) the absorption; (**b**) the transmission.

Polarization independence is critically important for the practical application of metamaterial absorbers. Figure 10 demonstrates the absorption and transmission coefficients of the metamaterial absorber under different polarization states when the VO_2_ film is at a high temperature (80 °C) and room temperature (27 °C). The results reveal that the ultra-broadband absorption characteristic and in-band transmission property of the metamaterial absorber exhibit polarization independence. These characteristics are of great importance for its practical application.

**Figure 11:**
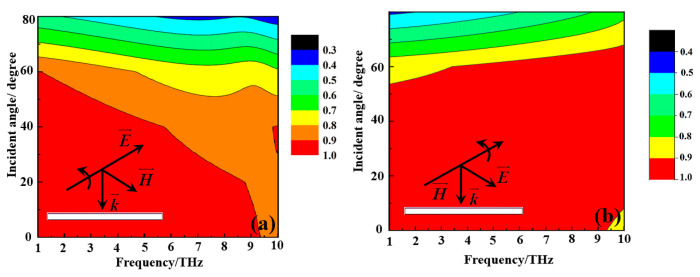
The absorption of the metamaterial absorber at various incident angles when the VO_2_ film is subjected to a high-temperature environment of 80 °C: (**a**) TE mode; (**b**) TM mode.

Figure 11 illustrates the absorption of the metamaterial absorber at various incident angles when the VO_2_ thin film is subjected to a high-temperature environment of 80 °C. As can be seen from Figure 11, at the TE mode, as the incident angle increases, the absorption bandwidth gradually decreases. However, within the range of 0–60°, the absorption in the 1–10 THz frequency range remains above 80%. The reason for the gradual reduction in the absorption bandwidth is that as the incident angle increases, the electric field component of the incident electromagnetic wave acting on the metamaterial absorber gradually decreases, leading to a weakening of its electric resonance. In the TM mode, within the incident angle range of 0–50°, the absorption in the 1–10 THz frequency range remains above 90%. Beyond 50°, as the incident angle increases, the absorption in the 1–10 THz frequency range gradually decreases, but within the range of 0–60°, the absorption in the 1–10 THz frequency range remains above 80%. The reason for the gradual reduction in the absorption bandwidth in TM mode is that as the incident angle increases, the magnetic field component of the incident electromagnetic wave acting on the metamaterial absorber gradually decreases, leading to a weakening of its magnetic resonance. The above results indicate that the ultra-wideband absorption characteristic of the metamaterial absorber is capable of maintaining high performance at large incident angles.

**Figure 12:**
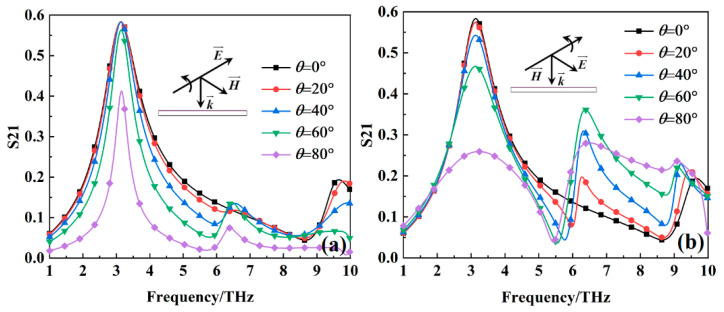
The transmission coefficients of the metamaterial absorber at different incident angles when the VO_2_ film is in a room-temperature environment (27 °C): (**a**) TE mode; (**b**) TM mode.

Figure 12 shows the transmission coefficients of the metamaterial absorber at different incident angles when the VO_2_ film is in a room-temperature environment (27 °C). As can be seen from the Figure 12, at the TE mode, the transmission coefficient at 3.1278 THz remains almost unchanged within the incident angle range of 0–60°. However, beyond 60°, the transmission coefficient gradually decreases. The primary reason for this is that as the incident angle increases, the electric field component of the incident electromagnetic wave acting on the metamaterial absorber gradually decreases. At the TM mode, as the incident angle increases, the transmission coefficient at 3.1278 THz gradually decreases. However, within the range of 0–40°, the transmission coefficient at 3.1278 THz remains above 50%. The main reason for this is that as the incident angle increases, the magnetic field component of the incident electromagnetic wave acting on the metamaterial absorber gradually decreases. These results indicate that the transmission characteristic of the metamaterial absorber also exhibits the capability to maintain performance at large incident angles.

**Figure 13:**
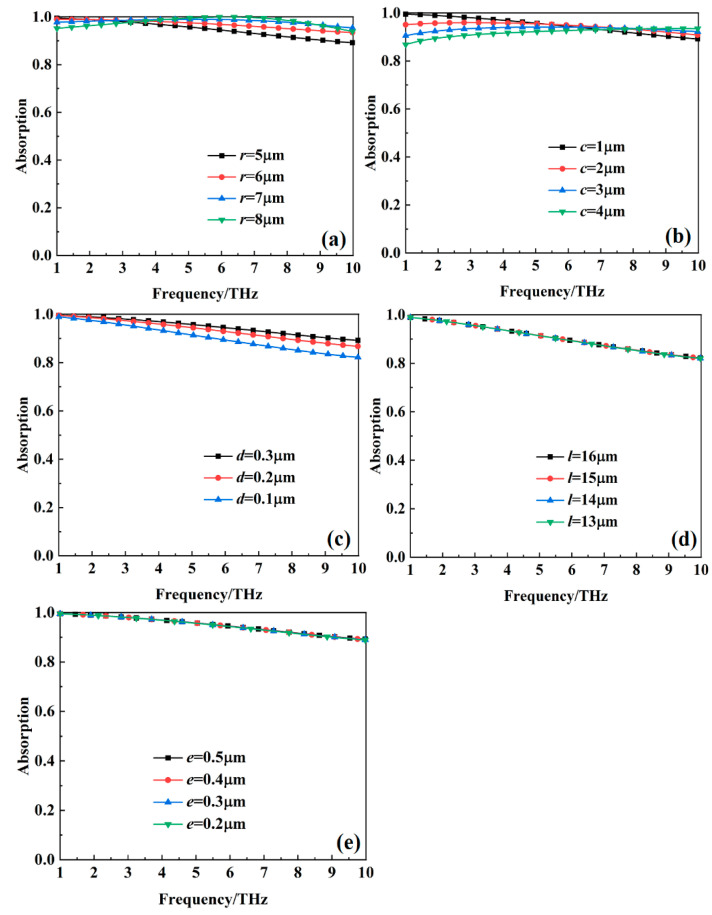
The absorption of the metamaterial absorber with different structural dimensional parameters when the VO_2_ thin film is at a high temperature (80 °C): (**a**) *r*; (**b**) *c*; (**c**) *d*; (**d**) *l*; (**e**) *e*.

Figure 13 shows the absorption of the metamaterial absorber with different structural dimensional parameters when the VO_2_ thin film is at a high temperature (80 °C). The results indicate that as dimension *c* increases and dimension *d* decreases, the absorption of the metamaterial gradually decreases. As the size parameter *r* increases, the bandwidth with an absorption over 90% remains essentially unchanged. On the other hand, the structural size parameters *l* and *e* have no effect on the absorption characteristic of the metamaterial absorber.

**Figure 14:**
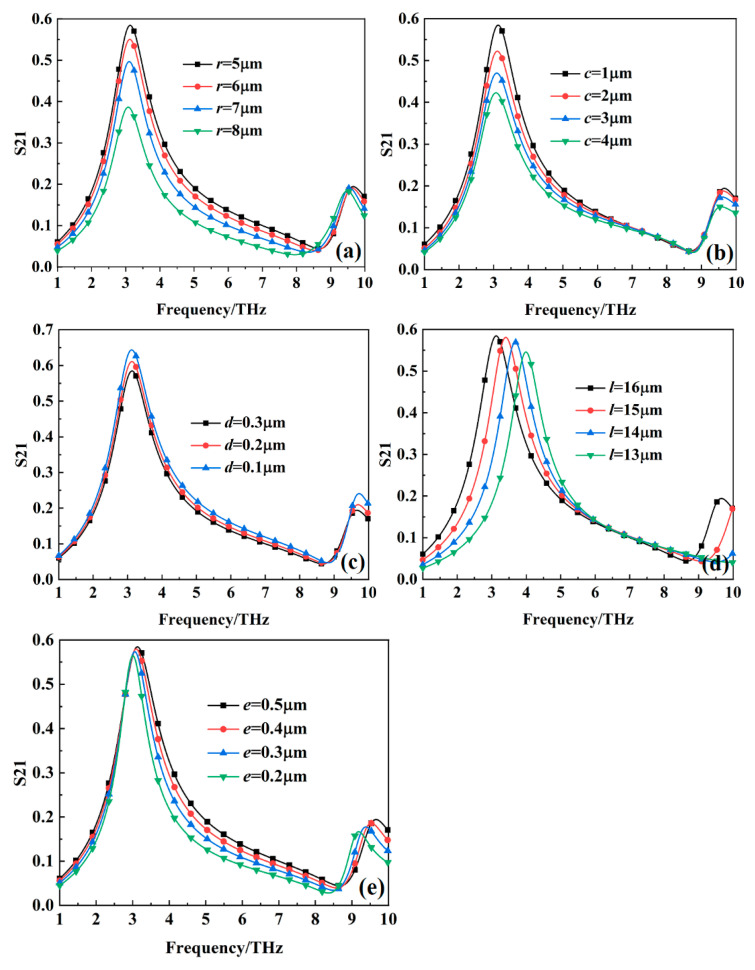
The transmission coefficient of the metamaterial absorber with different structural dimensional parameters when the VO_2_ thin film is at room temperature (27 °C): (**a**) *r*; (**b**) *c*; (**c**) *d*; (**d**) *l*; (**e**) *e*.

Figure 14 illustrates the transmission coefficient of the metamaterial absorber with different structural dimensional parameters when the VO_2_ thin film is at room temperature (27 °C). It can be observed that as the structural parameters *r*, *c*, and *d* increase, the transmission coefficient of the metamaterial absorber at 3.1278 THz gradually decreases. As parameter *l* decreases, the transmission frequency shifts toward higher frequencies and the transmission coefficient gradually decreases. Similarly, as parameter *e* decreases, the transmission frequency shifts toward lower frequencies and the transmission coefficient also gradually decreases.

## 4. Conclusions

In order to enhance the practical application of metamaterial absorbers, we have proposed and demonstrated a thermally controlled ultra-wideband wide incident angle metamaterial absorber with switchable transmission at the THz band, which is based on the thermally controlled conductivity of VO_2_ thin films. The proposed metamaterial achieves ultra-wideband absorption from 1 to 10 THz, corresponding to the VO_2_ thin film conductivity of 2 × 10^5^ S/m S/m at a high temperature (80 °C). Additionally, at room temperature (27 °C), the conductivity of VO_2_ thin films can be lowered to 20 S/m, which creates a transmission window for the incident wave at 3.1278 THz, with a transmission coefficient of 60%. The mechanism of wideband absorption is explained through monitoring and analysis of the surface current distribution. We also demonstrate that the ultra-wideband absorption and transmission property of the metamaterial absorber is polarization-insensitive and exhibits the capability to maintain performance at large incident angles. Our work provides enormous potential application value for stealth in communication equipment and radar systems at the THz band.

## Figures and Tables

**Table 1:** A comparison of the performance of our metamaterial absorber with the works in Refs. [23,24,25,26].

Metamaterial Absorber	Absorption Bandwidth	Transmission Coefficient	Fabrication Complexity
In Ref [23]	narrow-band absorption at 1.247 THz	90% at 0.48 THz	Easy to fabricate
In Ref [24]	Quad-band absorption at 0.66 THz, 1.22 THz, 2.14 THz, and 2.48 THz	81% at 0.9 THz	Easy to fabricate
In Ref [25]	Wideband absorption with the bandwidth of 5.8 THz	86% at 4.7 THz	Difficult to fabricate
In Ref [26]	Wideband absorption with the bandwidth of 5.11 THz	90% from 1 THz to 10 THz	Easy to fabricate
Our designed	Wideband absorption with the bandwidth of 5.11 THz	55% at 3.1278 THz	Easy to fabricate

## Data Availability

The data that support the findings of this study are available from the corresponding author upon reasonable request.
